# Anthracycline-based and gemcitabine-based chemotherapy in the adjuvant setting for stage I uterine leiomyosarcoma: a retrospective analysis at two reference centers

**DOI:** 10.1186/s13569-020-00139-3

**Published:** 2020-08-28

**Authors:** Giovanni Fucà, Chiara Fabbroni, Rosanna Mancari, Sara Manglaviti, Giorgio Bogani, Elena Fumagalli, Rossella Bertulli, Carlo Morosi, Paola Collini, Francesco Raspagliesi, Nicoletta Colombo, Paolo G. Casali, Roberta Sanfilippo

**Affiliations:** 1 Medical Oncology Unit 2, Medical Oncology Department, Fondazione IRCCS Istituto Nazionale Dei Tumori Di Milano, Milan, Italy; 2 Program of Gynecologic Oncology, IEO, Istituto Europeo Di Oncologia, IRCCS, Milan, Italy; 3 Department of Gynecologic Oncology, Fondazione IRCCS Istituto Nazionale Dei Tumori Di Milano, Milan, Italy; 4 Department of Radiology, Fondazione IRCCS Istituto Nazionale Dei Tumori Di Milano, Milan, Italy; 5 Department of Pathology, Fondazione IRCCS Istituto Nazionale Dei Tumori Di Milano, Milan, Italy; 6 University of Milan-Bicocca, Milan, Italy; 7 Oncology and Haemato-Oncology Department, University of Milan, Milan, Italy

**Keywords:** Uterine leiomyosarcoma, Adjuvant chemotherapy, Gemcitabine, Anthracycline

## Abstract

**Background:**

Radically resected early uterine leiomyosarcoma (eULMS) is still marked by a poor prognosis. Adjuvant strategies investigated up to now have not been corroborated by controlled studies. We retrospectively reviewed the clinical outcome of eULMS patients treated with adjuvant anthracycline-based or gemcitabine-based chemotherapy at two Italian reference centers.

**Methods:**

In this explorative, retrospective, cohort analysis, we included all the consecutive patients with radically resected eULMS treated at two centers between 1997 and 2017.

**Results:**

A total of 109 consecutive patients were included. Sixty-six (60%) received an anthracycline-based regimen, whereas 43 (40%) received a gemcitabine-based regimen. Median disease-free survival (DFS) was 41.3 months with anthracycline-based regimens compared to 20.9 months with gemcitabine-based regimens (HR: 0.49; 95% CI: 0.30–0.80; *P* = 0.004). In the multivariable model, anthracycline-based regimens were independently associated with a better DFS. No difference in terms of overall survival was observed.

**Conclusions:**

DFS was not the same by using an anthracycline-based or a gemcitabine-based adjuvant chemotherapy for patients with radically resected eULMS. The results of our study are in line with recent prospective controlled evidence in limb and superficial trunk soft tissue sarcomas. The role of anthracycline-based adjuvant chemotherapy should still be viewed as a research issue in eULMS.

## Background

Uterine leiomyosarcoma (ULMS) represents 1–2% of all uterine neoplasms. It is the most common type of uterine sarcomas, with an incidence of about 0.55/100,000 women per year [1]. Surgery is considered the mainstay treatment in uterine-confined ULMS (FIGO stage I) [2], avoiding procedures associated with a possible tumor spillage (i.e. morcellation) that are discouraged by guidelines because of the negative impact on patients' prognosis [3, 4]. Regrettably, ULMS is characterized by a poor prognosis even if diagnosed at an early stage [5]. Until now, unfortunately, adjuvant strategies investigated failed to be demonstrated to improve overall survival. Radiation therapy did not add any benefit to surgery alone in a large randomized trial [6], in spite of positive evidence provided by uncontrolled and retrospective studies, as far as the local regional relapse rate is concerned. The efficacy of adjuvant chemotherapy is still an open issue in soft-tissue sarcomas, and therefore in uterine leiomyosarcomas as well, in the absence of randomized study dedicated to uterine leiomyosarcoma but one, recently closed for low accrual. On the other hand, interesting progression-free survival was shown in patients treated with adjuvant chemotherapy by uncontrolled clinical studies [7, 8]. Intriguingly, in patients with localized somatic leiomyosarcoma (LMS), neoadjuvant anthracycline-based chemotherapy showed a superiority in terms of overall survival compared to gemcitabine-based chemotherapy in a randomized large study recently reported [9]. Thus, the role of adjuvant chemotherapy for early stage ULMS is still undefined [10]. In this paper, we report on a retrospective review of all the consecutive cases of uterine-confined ULMS treated with adjuvant chemotherapy at two Italian reference cancer centers, highlighting two groups, one treated with anthracycline-based and the other treated with gemcitabine-based regimens.

## Methods

### Patients population

This was an explorative, retrospective, cohort analysis. Institutional registries of two reference cancer centers in Italy (Fondazione IRCCS Istituto Nazionale dei Tumori, Milan; European Institute of Oncology, Milan) were searched in order to identify all consecutive patients with a histologic diagnosis of FIGO stage I uterine leiomyosarcoma treated with adjuvant chemotherapy between 1997 and 2017. The choice of regimen (anthracycline-based vs gemcitabine-based) was driven by the literature evidences available at the time of treatment initiation, the safety profile and patients’ preferences. Pathologic diagnosis was reviewed at each center by an expert pathologist according to the Stanford Criteria [11]. Data about demographics_,_ mitotic index, surgery, adjuvant chemotherapy, association with adjuvant radiotherapy and clinical outcomes were retrieved. Patients were included in two cohorts according to whether they received an anthracycline-based or a gemcitabine-based regimen.

The study was approved by the Institutional Review Boards of Fondazione IRCCS Istituto Nazionale dei Tumori di Milano and European Institute of Oncology and was conducted according to the ethical principles for medical research involving human subjects adopted in the Declaration of Helsinki. All the patients signed an informed consent for the use of their clinico-pathological data for research purposes.

### Statistical analyses

Disease-free survival (DFS) was defined as the time from surgical resection of uterine leiomyosarcoma to radiological evidence of recurrence or death from any cause. Overall survival (OS) was defined as the time from surgical resection of uterine leiomyosarcoma to death from any cause. Chi-square test, Fisher exact test or Mann–Whitney U test were used, as appropriate, to assess the association between clinico-pathological characteristics and type of adjuvant regimen and between relapse and type of adjuvant chemotherapy regimen. For survival analysis, we used the Kaplan–Meier method and Cox proportional hazards regression model. In Cox proportional hazards regression models, covariates with *P* < 0.1 in the univariable model were included in the multivariable model. Statistical significance was set at a threshold of *P* = 0.05. Statistical analyses were performed using R software (version 3.5.0) and RStudio software (version 1.1.453).

## Results

### Patients characteristics

Between 1997 and 2017, a total of 109 consecutive patients with resected stage I ULMS were treated with adjuvant chemotherapy at two Italian reference cancer centers. Sixty-six patients (60%) received an anthracycline-based regimen, whereas 43 (40%) received a gemcitabine-based regimen. Additional file 1: Table S1 shows the details about adjuvant chemotherapy regimens used. Patient and disease characteristics are summarized in Table 1. Median age was 50 years (interquartile range [IQR]: 43–57) and 83 out of 109 patients (81%) had a stage IB ULMS. Data about morcellation and bilateral oophorectomy at the time of surgery were available for 72 and 101 patients, respectively. Overall, 20 patients (28%) received morcellation and 74 (73%) underwent bilateral oophorectomy. Most of the patients (98 out of 109, i.e. 90%) did not receive adjuvant radiotherapy. Clinico-pathological characteristics were well balanced between the two cohorts except for morcellation, that was more frequent in patients receiving gemcitabine-based adjuvant chemotherapy as compared to patients receiving anthracycline-based adjuvant chemotherapy (42 vs 20%, respectively).

**Table 1:** Patients and disease characteristics in the entire population and according to adjuvant regimen

Characteristic	Total (N = 109)	G based (N = 43)	A based (N = 66)	P*
	N (%)	N (%)	N (%)	
Center				0.16
INT	78 (72)	34 (79)	44 (67)	
IEO	31 (28)	9 (21)	22 (33)	
Age				0.59
Median	50	49	52	
IQR	43–57	43–56	44–59	
Stage				0.59
IA	20 (19)	7 (17)	13 (21)	
IB	83 (81)	35 (83)	48 (79)	
NA	6	1	5	
Mitotic index				0.37
Median	21	25	16	
IQR	12–34	14–34	12–33	
NA	55	16	39	
Surgery				0.53
LPT	90 (88)	34 (85)	56 (90)	
LPS	12 (12)	6 (15)	6 (10)	
NA	7	3	4	
Morcellation				0.04
No	52 (72)	15 (58)	37 (80)	
Yes	20 (28)	11 (42)	9 (20)	
NA	37	17	20	
Oophorectomy				0.19
No	27 (27)	13 (34)	14 (22)	
Yes	74 (73)	25 (66)	49 (78)	
NA	8	5	3	
RT				0.05
No	98 (90)	42 (98)	56 (85)	
Yes	11 (10)	1 (2)	10 (15)	

*Chi-square test, Fisher exact test or Mann-Whitney U test as appropriate

*INT* Fondazione IRCCS Istituto Nazionale dei Tumori of Milan, *IEO* European Institute of Oncology, *IQR* interquartile range, *G* gemcitabine, *A* anthracycline, *RT* radiotherapy

### Clinical outcomes according to the adjuvant chemotherapy regimen

**Fig. 1:**
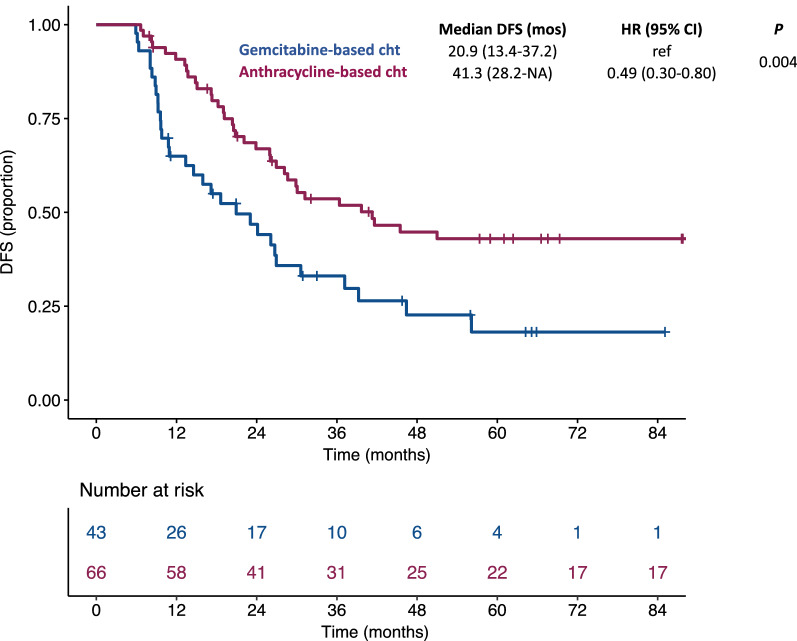
Kaplan–Meier curves for disease-free survival according to the adjuvant chemotherapy regimen

**Fig. 2:**
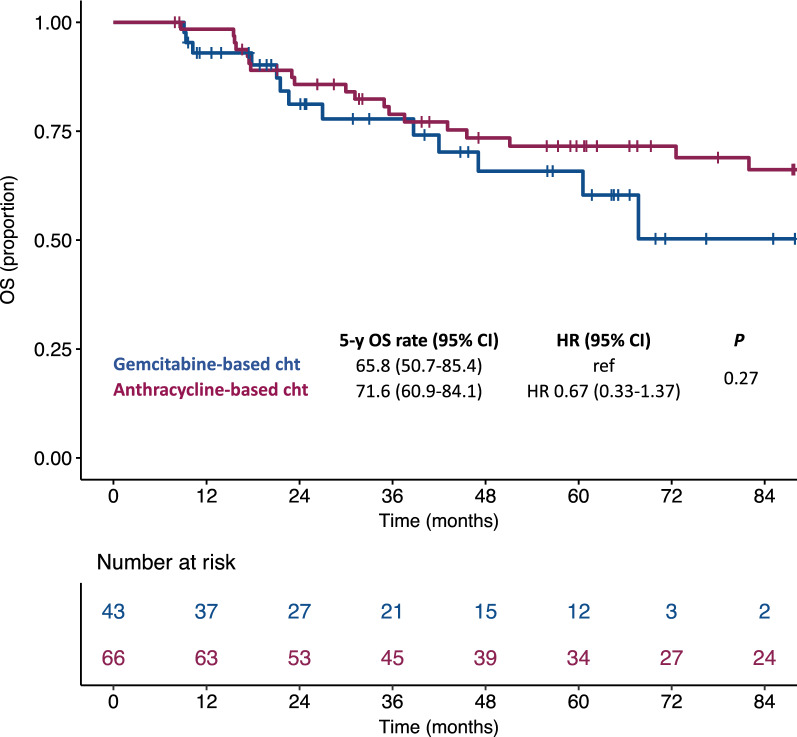
Kaplan–Meier curves for overall survival according to the adjuvant chemotherapy regimen

After a median follow up time of 87.8 months (IQR: 58.9–122.9), we observed a total of 38 relapses and 24 deaths. Specifically, we observed a relapse of disease in 38 patients treated with anthracycline-based adjuvant chemotherapy and 31 treated with gemcitabine-based adjuvant chemotherapy. Median DFS was 41.3 months (95% CI: 28.2-NA) for patients treated with anthracycline-based adjuvant chemotherapy (3-years DFS rate: 53.6%) compared to 20.9 months (95% CI: 13.4–37.2) for patients treated with gemcitabine-based adjuvant chemotherapy (3-years DFS rate: 33.1%) (HR: 0.49; 95% CI: 0.30–0.80; *P* = 0.004) (Fig. 1). In the multivariable model, including other covariates associated with DFS (i.e. stage and mitotic index), the use of anthracycline-based adjuvant chemotherapy independently correlated with a better DFS (HR: 0.37; 95% CI: 0.17–0.80; *P* = 0.01) (Table 2). No difference in terms of overall survival was found between patients treated with anthracycline-based adjuvant chemotherapy and patients treated with gemcitabine-based adjuvant chemotherapy (5-years OS rate: 71.6% vs 65.8%, respectively; HR: 0.67; 95% CI: 0.33–1.37; *P* = 0.27) (Fig. 2). When we analyzed the effect of the two different adjuvant regimens according to FIGO stage, we observed a significant advantage in terms of DFS for anthracycline-based regimens in patients with stage IB (HR: 0.62; 95% CI: 0.17–2.32) ULMS, but not in patients with stage IA (HR: 0.42; 95% CI: 0.24–0.72) even if the interaction test was not statistically significant (*P* = 0.77) (Additional file 2: Figure S1). Accordingly, a non-significant trend toward a better OS with anthracycline-based adjuvant chemotherapy was observed in patients with stage IB ULMS (Additional file 2: Figure S1).

**Table 2:** Cox proportional hazard regression models for disease-free survival

Characteristic	Univariable model		Multivariable model	
	HR (95% CI)	*P*	HR (95% CI)	*P*
Age (years)	1.07 (0.84–1.35)^b^	0.59	–	–
Stage				
IB vs IA	1.99 (0.98–4.03)	0.06	4.27 (1.28–14.20)	0.02
Mitotic index				
^a^34 vs 12	1.02 (1.00–1.03)	0.07	1.01 (0.99–1.03)	0.22
Surgery				
LPS vs LPT	0.82 (0.35–1.89)	0.64	–	–
Morcellation				
Yes vs No	1.01 (0.51–2.01)	0.98	–	–
Oophorectomy				
Yes vs No	0.92 (0.53–81.60)	0.78	–	–
RT				
Yes vs No	0.68 (0.29–1.58)	0.37	–	–
Regimen				
A based vs G based	0.49 (0.30–0.80)	0.004	0.37 (0.17–0.80)	0.01

^a^The reported values are the third and first quartiles of the variable distribution

^b^Hazard ratio for a 10 years increase in age

*HR* hazard ratio, *CI* confidence interval, *G* gemcitabine, *A* anthracycline, *RT* radiotherapy

## Discussion

In this explorative, retrospective cohort analysis of 109 consecutive patients with completely resected stage I ULMS treated with adjuvant chemotherapy, we observed a different benefit, especially in terms of DFS, for patients treated with anthracycline-based chemotherapy compared to patients treated with gemcitabine-based chemotherapy, even after adjusting for other relevant variables (i.e. FIGO stage and mitotic index).

Today, the standard of care of localized early stage ULMS is radical surgery alone [3, 4, 12]. Unfortunately, after the failure for low accrual of the first large randomized study of adjuvant chemotherapy versus observation, it is very unlikely that a new controlled international study will be proposed to establish a definitive role of the adjuvant chemotherapy in ULMS. On the other hand, two phase 2 studies of adjuvant chemotherapy, with gemcitabine and docetaxel and with gemcitabine and docetaxel followed by anthracycline, respectively, showed an interesting DFS for adjuvant chemotherapy compared with historical control [7, 8]. In particular, the addition of four cycles of anthracycline to gemcitabine and docetaxel demonstrated an improvement in DFS in respect of the use of gemcitabine and docetaxel alone previously reported [8]. At the same time, a recent controlled study of neo-adjuvant tailored chemotherapy versus anthracycline-based chemotherapy in high-risk soft tissue sarcoma of limbs and superficial trunk, including somatic leiomyosarcomas, showed better results for patients treated with an anthracycline-based regimen compared to a histology-driven one (which was based on gemcitabine for leiomyosarcomas).

This case series analysis is retrospective in nature and included patients treated in a long-time span (20 years) with different schedules, although they were all managed at two reference centers for gynecological sarcomas.

## Conclusion

In conclusion, there is still a lack of unequivocal evidence on the survival advantage in using adjuvant chemotherapy for high-risk soft tissue sarcoma (including ULMS), despite the meta-analysis by the Sarcoma Meta-analysis Collaboration showed a possible small benefit [13]. Histotype-tailored chemotherapy is not the answer, as has been clearly previously demonstrated in the neoadjuvant setting [9] and corroborated by the present adjuvant data. Indeed, our results provide further evidence, in addition to the ISG-STS 1001 trial, on the value of anthracycline-based chemotherapy in ULMS. Given the heterogeneity of sarcomas, we probably need focused prospective trials powered for the individual tumor types (such as ULMS), which is impossible to achieve without international collaborations. Based on the results of the present analysis and literature data, we believe that an optimal design for a prospective trial should include a stage I ULMS cohort using anthracycline-based adjuvant chemotherapy and should be powered for separate subgroup analyses of IA and IB disease.

## Supplementary information

**Additional file 1: Table S1.** Specific adjuvant chemotherapy regimens.

**Additional file 2: Figure S2.** Kaplan–Meier curves for disease-free survival and overall survival according to stage (panels A and B, respectively).

## Data Availability

The datasets generated during and/or analyzed during the current study are available from the corresponding author on reasonable request.

## Abbreviations

ULMS: Uterine leiomyosarcoma
eULMS: Early uterine leiomyosarcoma
IQR: Interquartile range
DFS: Disease-free survival
OS: Overall survival
