# Supplementary material for: Anthracycline-based and gemcitabine-based chemotherapy in the adjuvant setting for stage I uterine leiomyosarcoma: a retrospective analysis at two reference centers
Source: Clin Sarcoma Res. 2020 Aug 28;10:17. doi: 10.1186/s13569-020-00139-3 (PMC7456084; doi:10.1186/s13569-020-00139-3)
Supplement: Supplementary file 1 — Additional file 1: Table S1. Specific adjuvant chemotherapy regimens. [file 13569_2020_139_MOESM1_ESM.pdf]

Additional Table 1. Specific adjuvant chemotherapy regimens.

Gemcitabine-based adjuvant chemotherapy	Total (N=43) N (%)
Gemcitabine plus docetaxel	40 (93)
Gemcitabine plus dacarbazine	3 (7)

Athracycline-based adjuvant chemotherapy	Total (N=66) N (%)
Doxorubicin	2 (3)
Epirubicin	2 (3)
Doxorubicin plus ifosfamide	8 (12)
Epirubicin plus ifosfamide	50 (76)
Doxorubicin plus dacarbazine	4 (6)
