# Supplementary material for: Anthracycline-based and gemcitabine-based chemotherapy in the adjuvant setting for stage I uterine leiomyosarcoma: a retrospective analysis at two reference centers
Source: Clin Sarcoma Res. 2020 Aug 28;10:17. doi: 10.1186/s13569-020-00139-3 (PMC7456084; doi:10.1186/s13569-020-00139-3)

Additional Figure 1. Kaplan-Meier curves for disease-free survival and overall survival according to stage (panels A and B, respectively).

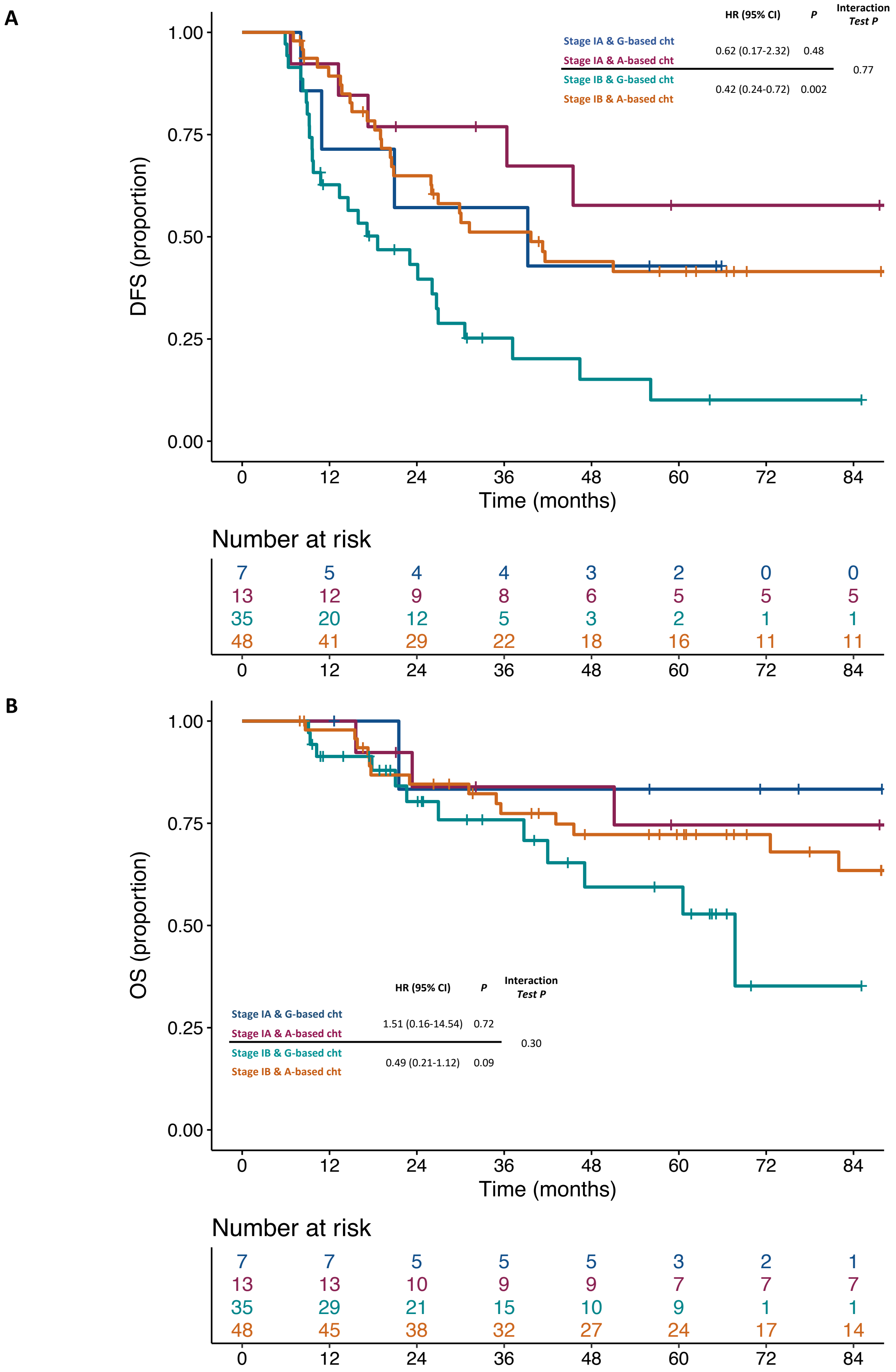

Supplement: Supplementary file 2 — Additional file 2: Figure S2. Kaplan–Meier curves for disease-free survival and overall survival according to stage (panels A and B, respectively). [file 13569_2020_139_MOESM2_ESM.pdf]
